# Biological therapies in monogenic autoinflammatory diseases: long-term efficacy and safety

**DOI:** 10.1186/1824-7288-40-S1-A26

**Published:** 2014-08-11

**Authors:** Maria A Pelagatti, Alessandro Cattoni, Carmelo Rizzari

**Affiliations:** 1 Department of Pediatrics, University of Milano-Bicocca, San Gerardo Hospital, 20900 Monza (MB), Italy

## Introduction

Autoinflammatory diseases (AIDs) represent a group of monogenic disorders, related to mutations in genes encoding proteins involved in the regulation of inflammatory responses. The aims of therapy in AIDs patients are controlling disease activity, improving patient’s quality of life and preventing long-term complications. Biological agents have recently changed the natural history and the prognosis of AIDs. According to a pathogenetic criterium, they can be classified as:

1 ) Anti-TNF agents: i) Etanercept, a dimeric fusion protein of TNFR2 linked with the Fc region of human IgG1, ii) Adalimumab, a fully human monoclonal anti-TNF antibody, iii) Infliximab, a mouse/human chimeric monoclonal anti-TNF antibody [1,2].

2) Anti-IL-1 agents: i) Anakinra, a competitive IL-1 receptor antagonist, ii) Canakinumab, a fully humanized anti-IL-1 monoclonal antibody, iii) Rilonacept, a fusion protein of IL-1 extracellular domains and Fc region of human IgG [1].

3) Anti-IL-6 agents: Tocilizumab, a monoclonal antibody binding and inhibiting both soluble and membrane IL-6 receptor [1,2].

## Clinical use of biological drugs in autoinflammatory disorders

→ **Familiar Mediterranean Fever (FMF)** is the only autoinflammatory disease in which a “classical” immunomodulator (colchicine) still plays a central role [3]. Nonetheless, a relevant number of non complete responders has been recently observed. According to the pivotal role of IL-1 in the pathogenesis of FMF [4,5], anti-IL-1 treatment has been employed in colchicine-resistant patients, with good response in terms of control of disease activity and systemic impairment [6,7].

→ In **TRAPS** (TNF Receptor Associated Periodic Fever Syndrome) patients, the failure of Infliximab and Adalimumab in controlling inflammatory flares has been already described [8]. Etanercept does not completely normalize symptoms or acute phase reactants [9] and the progressive development of neutralizing antibodies may compromise its effectiveness [2]. On the other hand, recent papers have shown the dramatic and persistent efficacy of anti-IL-1 treatment in TRAPS patients [10,11].

→ In **CAPS** (Cryopirin Associated Periodic Syndromes) patients treated with Anakinra, the clinical improvement appears to be strictly dependent on continuous drug administration [12,13]. Moreover, preliminary results on Rilonacept and Canakinumab seem to be promising [14-16].

→ In **Hyper-IgD** (Mevalonate Kinase Deficiency), Anakinra has shown to be markedly effective in different case reports, while Etanercept provided variable results [2].

## Conclusions

Although biologic agents are related to frequent side effects, their administration has revolutionized the clinical approach to AIDs patients. Further trials are needed to definitively assess their safety and efficacy.
